# A new method for concomitant evaluation of drug combinations for their antimicrobial properties

**DOI:** 10.1016/j.mex.2024.103122

**Published:** 2024-12-22

**Authors:** Syed Ahmed Rizvi, Kaniz F Chowdhury, Chiara Borsetto, Emma R Travis, Mohammad Saif, Vikar Ahmed, Laura J Carter, Iqbal Ahmad, Alan McNally, Qazi Mohd Rizwanul Haq

**Affiliations:** a Department of Biosciences, Jamia Millia Islamia, New Delhi, India; b School of Geography, Faculty of Environment, University of Leeds, Leeds, UK; c School of Life Sciences, University of Warwick, Coventry, United Kingdom; d Department of Agricultural Microbiology, Aligarh Muslim University, Aligarh, 202002, India; e Institute of Microbiology and Infection, College of Medical and Dental Sciences, University of Birmingham, Birmingham, UK

**Keywords:** Antimicrobial resistance (AMR), Biofilm, Minimum inhibitory concentration (MIC), Concomitant evaluation of drug combinations for their antimicrobial properties

## Abstract

Microbial pathogens have developed resistance mechanisms to almost every antibiotic available. There is a need to synthesize or screen new natural compounds to combat the development of drug-resistant pathogens. One of the commonly used methods to evaluate the antimicrobial activity of two or more antibiotics involves a checkerboard assay, which is cumbersome, time-consuming, and expensive. We have developed a quick, reliable, and cost-effective method to evaluate the antimicrobial effect of two or more antibiotics at fixed doses with different concentrations of a novel natural ingredient or test compound.

The technique involves the following steps:

• Preparation of a bacterial culture of the test strain at 0.5 McFarland standard (0.1 OD at 600 nm), and preparation of stock solutions for the chemical of interest and standard drugs.
• The required amount of all three components can be dispensed into respective wells of a microplate using multichannel pipette.
• Optical density (OD) values obtained would be directly related to the individual as well as combined effect of compounds on the given bacterial strain.

## Graphical abstract

**Figure:**
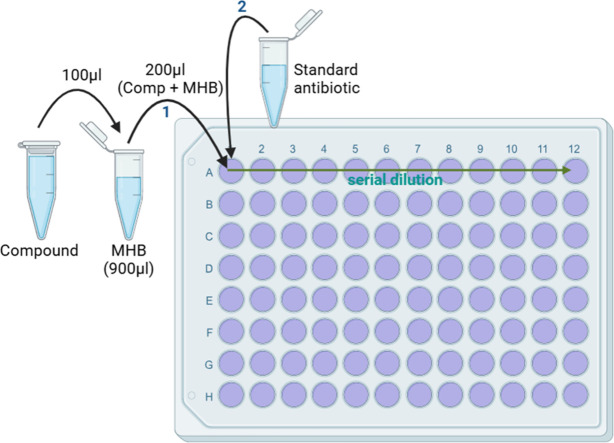

Specifications table

**Table:** 

Subject area:	Pharmacology, Toxicology and Pharmaceutical Science
More specific subject area:	Antimicrobial activity/ Biofilm/ MIC
Name of your method:	Concomitant evaluation of drug combinations for their antimicrobial properties
Name and reference of original method:	Checkerboard analysis assay
Resource availability	Microtiter plate, multichannel pipette, Bacterial growth media, Reagents (Antibiotics/ Test compound/ crystal violet), and microplate reader.

## Background

**Fig. 1:**
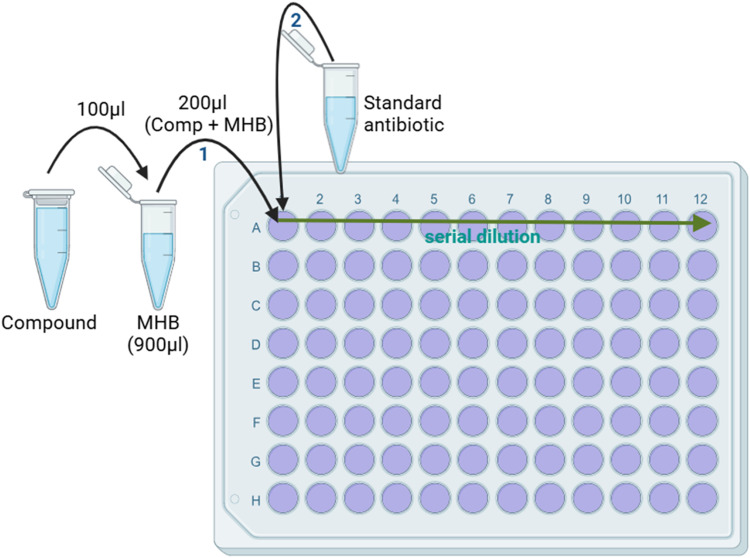
Graphical representation of the procedure.

Due to the rapid increase in infections caused by antimicrobial-resistant (AMR) pathogens, it is estimated that nearly 10 million premature deaths per year will occur by 2050 [1]. Therefore, combating AMR by developing new antibiotics, combination therapy, and novel therapeutic approaches is imperative. Due to the scarcity of new antibiotics and the presence of multiple antibiotic-resistance genes on a plasmid, combination antibiotic therapy has been recently introduced as a method of choice for the treatment of infections caused by resistant bacteria [2]. The currently available protocol uses checkerboard analysis to determine the combined effect of two antibiotics [3,4]. In the case of three or more compounds, the number of combinations will increase drastically, which is time-consuming. The developed method is efficient and determines the combined effect of two or more antibiotics at fixed doses with different concentrations of a novel natural ingredient or test compound (Fig. 1).

The application of the proposed method could include:

1. This method can determine the MIC and inhibition/ eradication of biofilm by alternative medicines/ natural antioxidants/ free radical initiators/ osmolytes in the presence of standard antibiotics.

## Method details

**Day 1 (For Biofilm formation assay)**

1. Prepare 1.33 times concentrated Tryptic Soy Broth (TSB) media and sterilise.
2. Depending upon the number of standard antibiotics, stock solutions of the antibiotics are prepared as:
  Stock concentration of standard drug (S_s_) = 20 x a x n (µg/ml)
  where, a: required concentration of standard antibiotic (µg/ml)
3. Make a stock solution of the test compound (to be serially diluted) in sterile saline water or appropriate solvent as if “b” µg/ml is required in the first well:
  Stock concentration of test compound/ drug (S_t_) = 40 x b (µg/ml) e.g., if there are two standard antibiotics to be tested at concentrations of 4 and 32 µg/ml along with a test compound at a starting concentration of 512 µg/ml, the values of a, b, S_s_, S_t_ will be: a_1_ = 4 a_2_ = 32 µg/ml b = 512 µg/ml
  S_s1_ = 20 × 4 × 2 = 160 µg/ml
  S_s2_ = 20 × 32 × 2 = 1280 µg/ml
  S_t_ = 40 × 512 = 20,480 µg/ml
4. Inoculate fresh TSB media with a single colony (pure culture) of the test isolate and incubate overnight at appropriate temperature.

**Day 2 (For Biofilm formation assay)**

5. Add 50 µl of TSB in all the required columns and 90 µl of TSB in the first column.
6. Add 10 µl of the stock solution of the test compound in the first column and serially dilute the test compound.
7. To evaluate the activity of standard antibiotic, the stock solution is diluted in sterile saline water or appropriate solvent to obtain final concentration of “b” µg/ml:
  $$\text{Volume}\;\text{of}\;\text{standard}\;\text{stock}\;\text{solution}\;\text{in}\;\text{the}\;\text{cocktail}\left(v_{s}\right)=\frac{\text{volume}\;\text{of}\;\text{total}\;\text{solution}}{5\text{xn}}$$
  If we make the final cocktail of 1200 µl, then add 120 µl of each antibiotic in 1080 µl of sterile saline water or appropriate solvent.
8. Add 50 µl of the cocktail in the respective wells to achieve the desired concentrations of the individual standard antibiotics.
9. Add 100 µl of 1:1 diluted test culture (with TSB) into each well.
10. Seal the plate and incubate at 37°C for 48 hours without shaking.

**Day 4 (For Biofilm formation assay)**

11. Decant media and wash the wells thrice with 200 µl 1x PBS (pH 7.4).
12. Add 200 µl of 0.1% CV solution in each well and incubate for 30 minutes at room temperature.
13. Wash the wells twice with PBS and add 200 µl of 95% ethanol.
14. Record the absorbance of the solution at 590 nm to evaluate the antimicrobial activity.
15. Compare the OD values obtained with the controls (strong biofilm former as a +ve control and TSB alone as a -ve control).

**Day 1 (For Minimum Inhibitory Concentration)**

1. Prepare 2 times concentrated Muller Hinton Broth (MHB) media and sterilise.
2. Incubate 1:300 volume of overnight grown culture into the fresh MHB media to get 0.5 McFarland growth (0.1 OD at 600 nm).
4. Depending upon the number of test compounds, stock solutions of standard antibiotics are prepared as:
  Stock concentration of standard antibiotic (S_s_) = 10 x a x n (µg/ml), where a: concentration of antibiotic required (µg/ml), n: No. of standard antibiotics.
5. Make a stock solution of the test compound (to be serially diluted) as:
  Stock concentration (s_t_) = 20 x b (µg/ml), where b: desired concentration of test compound in the first well. e.g., if there are two standard antibiotics to be tested at concentrations of 4 and 32 µg/ml along with a test compound at a starting concentration of 512 µg/ml, the values of a, b, S_s_, S_t_ will be: a_1_ = 4 µg/ml a_2_ = 32 µg/ml b = 512 µg/ml
  S_s1_ = 10 × 4 × 2 = 80 µg/ml
  S_s2_ = 10 × 32 × 2 = 640 µg/ml
  S_t_ = 20 × 512 = 10,240 µg/ml
6. Add 50 µl of Muller Hinton broth in all the required columns and 90 µl of MHB in the first column.
7. Add 10 µl of the stock solution of the test compound in the first column and serially dilute the test compound.
8. To evaluate the activity of standard antibiotic, the stock solution is diluted in sterile double distilled water or appropriate solvent to obtain final concentration of “b” µg/ml:
  $$\text{Volume}\;\text{of}\;\text{standard}\;\text{stock}\;\text{solution}\;\text{in}\;\text{the}\;\text{cocktail}\left(v_{s}\right)=\frac{\text{volume}\;\text{of}\;\text{total}\;\text{solution}}{5\text{xn}}$$
  If we make the final cocktail of 1200 µl, then add 120 µl of all three antibiotics in 1080 µl of sterile double distilled water or appropriate solvent.
9. Add 50 µl of the cocktail in the respective wells to achieve the desired concentrations of the individual standard antibiotics.
10. Add 10 µl of 1:100 diluted secondary bacterial culture in all the wells.
11. Incubate the plate for 16–20 hours, depending on the bacteria.
12. Record the absorbance at 600 nm to determine the MIC in the microplate reader.

## Method validation

Fresh MHB media was transferred into each well, then chloramphenicol was serially diluted with a starting concentration of 100 µg/ml. Add trimethoprim and kanamycin in each well to achieve final concentrations of 0.02 and 0.3 µg/ml, respectively. 10 µl of bacterial culture having the OD value of 0.1 was transferred into each well of the 96-wells microplate. After incubating for 14–18 hours at 37°C, OD values were measured at 600 nm. No Change in OD values (MIC_89_) was observed, and thus, the compound has no correlation with the antibiotics at the aforementioned fixed doses.

**Table:** 

Trimethoprim	Kanamycin	Chloramphenicol	Combination (All three)
MIC_12_ (0.3 µg/ml)	MIC_12_ (0.3 µg/ml)	MIC_89_ (6.25 µg/ml)	MIC_89_ (6.25 µg/ml)

In MIC_x_,

$$x=\frac{\left(\text{Control}\;\text{OD}-\text{test}\;\text{OD}\right)}{\text{Control}\;\text{OD}}\times 100$$

Fresh MHB media (100 µl) was transferred into each well, and then sulfamethoxazole was serially diluted with a starting concentration of 100 µg/ml. Trimethoprim and kanamycin were added into each well to achieve final concentrations of 0.06 and 0.98 µg/ml, respectively. The bacterial culture (10 µl) having 0.5 McFarland standard was transferred into each well of the 96-wells microplate. After incubating for 14–18 hours at 37°C, OD values were measured at 600 nm. Changes observed in OD values were significant (MIC_89_); thus, the compound in combination with antibiotics has antimicrobial activity. There was no change in the OD values with either compound or antibiotic alone.

**Table:** 

Trimethoprim	Kanamycin	Sulfamethoxazole	Combination
MIC_8_ (0.06 µg/ml)	MIC_32_ (0.98 µg/ml)	MIC_11_ (0.19 µg/ml)	MIC_89_ (0.19 µg/ml)

## Limitations

Only one test compound/ drug could be tested at different concentrations with two or more standard antibiotics.

## Ethics statements

N/A.

## CRediT authorship contribution statement

**Syed Ahmed Rizvi:** Conceptualization, Methodology, Validity tests, Writing- Original draft preparation, and data curation. **KF Chowdhury:** Conceptualization and methodology. **Chiara Borsetto:** Conceptualization and methodology. **Emma R Travis:** Conceptualization and methodology. **Mohammad Saif:** Validity tests. **Vikar Ahmed:** Validity tests and investigations. **LJ Carter:** Reviewing and Editing. **I Ahmad:** Reviewing and Editing. **A McNally:** Reviewing and Editing. **Qazi Mohd Rizwanul Haq:** Supervision, Writing- Reviewing and Editing, and resources.

## Declaration of competing interest

The authors declare that they have no known competing financial interests or personal relationships that could have appeared to influence the work reported in this paper.

## Data Availability

No data was used for the research described in the article.
